# Smartphone Pupillometry and Machine Learning for Detection of Acute Mild Traumatic Brain Injury: Cohort Study

**DOI:** 10.2196/58398

**Published:** 2024-06-13

**Authors:** Anthony J Maxin, Do H Lim, Sophie Kush, Jack Carpenter, Rami Shaibani, Bernice G Gulek, Kimberly G Harmon, Alex Mariakakis, Lynn B McGrath, Michael R Levitt

**Affiliations:** 1 Department of Neurological Surgery University of Washington Seattle, WA United States; 2 School of Medicine Creighton University Omaha, NE United States; 3 Stroke & Applied Neuroscience Center University of Washington Seattle, WA United States; 4 Department of Neurological Surgery Weill Cornell Medicine New York, NY United States; 5 Santa Clara University Santa Clara, CA United States; 6 Department of Psychiatry & Behavioral Sciences Stanford University Stanford, CA United States; 7 Department of Family Medicine University of Washington Seattle, WA United States; 8 Department of Computer Science University of Toronto Toronto, ON Canada; 9 Department of Radiology University of Washington Seattle, WA United States; 10 Department of Mechanical Engineering University of Washington Seattle, WA United States

**Keywords:** smartphone pupillometry, pupillary light reflex, biomarkers, digital health, mild traumatic brain injury, concussion, machine learning, artificial intelligence, AI, pupillary, pilot study, brain, brain injury, injury, diagnostic, pupillometer, neuroimaging, diagnosis, artificial, mobile phone

## Abstract

**Background:**

Quantitative pupillometry is used in mild traumatic brain injury (mTBI) with changes in pupil reactivity noted after blast injury, chronic mTBI, and sports-related concussion.

**Objective:**

We evaluated the diagnostic capabilities of a smartphone-based digital pupillometer to differentiate patients with mTBI in the emergency department from controls.

**Methods:**

Adult patients diagnosed with acute mTBI with normal neuroimaging were evaluated in an emergency department within 36 hours of injury (control group: healthy adults). The PupilScreen smartphone pupillometer was used to measure the pupillary light reflex (PLR), and quantitative curve morphological parameters of the PLR were compared between mTBI and healthy controls. To address the class imbalance in our sample, a synthetic minority oversampling technique was applied. All possible combinations of PLR parameters produced by the smartphone pupillometer were then applied as features to 4 binary classification machine learning algorithms: random forest, k-nearest neighbors, support vector machine, and logistic regression. A 10-fold cross-validation technique stratified by cohort was used to produce accuracy, sensitivity, specificity, area under the curve, and *F*_1_-score metrics for the classification of mTBI versus healthy participants.

**Results:**

Of 12 patients with acute mTBI, 33% (4/12) were female (mean age 54.1, SD 22.2 years), and 58% (7/12) were White with a median Glasgow Coma Scale (GCS) of 15. Of the 132 healthy patients, 67% (88/132) were female, with a mean age of 36 (SD 10.2) years and 64% (84/132) were White with a median GCS of 15. Significant differences were observed in PLR recordings between healthy controls and patients with acute mTBI in the PLR parameters, that are (1) percent change (mean 34%, SD 8.3% vs mean 26%, SD 7.9%; *P*<.001), (2) minimum pupillary diameter (mean 34.8, SD 6.1 pixels vs mean 29.7, SD 6.1 pixels; *P*=.004), (3) maximum pupillary diameter (mean 53.6, SD 12.4 pixels vs mean 40.9, SD 11.9 pixels; *P*<.001), and (4) mean constriction velocity (mean 11.5, SD 5.0 pixels/second vs mean 6.8, SD 3.0 pixels/second; *P*<.001) between cohorts. After the synthetic minority oversampling technique, both cohorts had a sample size of 132 recordings. The best-performing binary classification model was a random forest model using the PLR parameters of latency, percent change, maximum diameter, minimum diameter, mean constriction velocity, and maximum constriction velocity as features. This model produced an overall accuracy of 93.5%, sensitivity of 96.2%, specificity of 90.9%, area under the curve of 0.936, and *F*_1_-score of 93.7% for differentiating between pupillary changes in mTBI and healthy participants. The absolute values are unable to be provided for the performance percentages reported here due to the mechanism of 10-fold cross validation that was used to obtain them.

**Conclusions:**

In this pilot study, quantitative smartphone pupillometry demonstrates the potential to be a useful tool in the future diagnosis of acute mTBI.

## Introduction

The pupillary light reflex (PLR) is a biomarker of neurological disease demonstrated by the reaction of the pupil to a light stimulus [1] that is commonly used in the management of moderate to severe traumatic brain injury (TBI) [2,3]. The pupil has both sympathetic and parasympathetic innervation that can be affected by mild TBI (mTBI). Traditional PLR assessment uses a manual penlight [4]; however, this method experiences poor interrater reliability, is highly subjective, and is of little use outside of moderate to severe TBI [4,5]. More recently, quantitative measurement of the PLR has been used as a biomarker for mTBI wherein the pupils are reactive but abnormal in a manner that is not easily detectable to the human eye [6]. Quantitative pupillometry is typically performed in the intensive care unit or in neuro–intensive care unit settings with United States Food and Drug Administration (FDA)–approved equipment (NeurOptics). There has been recent interest in the use of this same equipment for the diagnosis of concussion in military personnel after the blast injury [7], to document pupillary changes in those with chronic mTBI [8,9], and most recently interest in the diagnosis of sports-related concussions [10].

We developed a smartphone quantitative pupillometry app (PupilScreen) that measures the PLR with greater accuracy and higher interrater reliability than the manual penlight [11]. This study aims to investigate the ability of the smartphone pupillometry app to differentiate between participants with acute mTBI (<36 hours after injury) and healthy controls.

## Methods

### Recruitment

We used a previously developed binocular smartphone pupillometer (PupilScreen), which quantifies PLR curve morphological parameters (Textbox 1) to examine differences in pupillary reactivity between participants with acute mTBI and healthy participants. The smartphone pupillometry app requires a standard iPhone (Apple) camera without external hardware and is connected to a cloud-based neural network computer vision algorithm [11-15]. The app interface includes an augmented reality screen overlay with eye holes that helps to standardize the distance from the phone to the pupils for each measurement [13]. Using this technique in previous studies, the median error of pupil detection to the ground truth pupil diameter in millimeters was 0.23 and the mean absolute relative percent difference between sequential measurements was mean 5.8% (SD 3%) [12].

Patients with a clinical diagnosis of acute mTBI were enrolled prospectively through availability sampling (as this was an exploratory pilot study) in an emergency department after presenting with head trauma and known mechanism of injury less than 36 hours post injury from July 2022 to March 2023. mTBI was defined according to the American College of Rehabilitation Medicine (ACRM) criteria [16]. Participants were excluded if they had any intracranial abnormalities on neuroimaging. A separate cohort of healthy participants was enrolled from hospital staff using availability sampling over the same time period, which excluded those with self-reported known neurological disease or recent history of TBI.

**Definitions of pupillary light reflex parameters.**

Latency (seconds [s]): time from onset of light stimulus to initial pupillary constriction

Percent change (%): percent change in pupillary diameter from maximum to minimum

Minimum pupillary diameter (pixels [px]): minimum diameter after light stimulus

Maximum pupillary diameter (px): average resting diameter before light stimulus

Mean constriction velocity (px/s): the average speed at which the pupil constricts after the light stimulus until the minimum diameter is reached

Maximum constriction velocity (px/s): the maximum speed at which the pupil constricts after the light stimulus until the minimum diameter is reached

Mean dilation velocity (px/s): the average speed at which the pupil dilates after removal of the light stimulus

### Statistical Analysis

The PLR parameters were averaged for each subject between the left and right eyes before analysis. Differences in PLR parameters between cohorts were examined using a one-tailed *t* test for independent means. A *P* value of <.05 was considered statistically significant and a post hoc Bonferroni correction was implemented to control the probability of committing a type I error in the results. In addition, an analysis was performed to demonstrate the classification ability of the PLR parameters as feature inputs to machine learning models in the task of differentiating between the healthy and mTBI cohorts. Due to the significant class imbalance present, a synthetic minority oversampling technique (SMOTE) [17] was used to oversample the mTBI cohort PLR parameters to match the sample size of the healthy cohort. All PLR parameters were analyzed using 4 separate binary classification machine learning models: random forest, k-nearest neighbors, logistic regression, and support vector machine [18]. A 10-fold cross-validation stratified by cohort (which respects the independence of the training and testing sets) was used to produce the following model performance metrics, that are overall accuracy, sensitivity, specificity, area under the curve (AUC), and *F*_1_-score, on the unseen test data sets. We report the best-performing feature combinations for each model type, based on AUC value, in differentiating PLR curves of patients with mTBI from healthy controls.

### Ethical Considerations

This study was approved by the University of Washington institutional review board (#8009), and an informed consent process was followed for all participants as approved by the institutional review board.

## Results

### Cohort Characteristics

A total of 12 patients diagnosed with mTBI and 132 healthy participants were enrolled. Subject demographics are listed in Table 1 and characteristics of their injury are listed in Multimedia Appendix 1. Participants with acute mTBI were studied for an average of 6.8 (range 0.5-29) hours after injury. A total of 10 out of 12 in this sample had a loss of consciousness (<30 minutes) and 10 out of 12 had posttraumatic amnesia. Mechanisms of injury included motor vehicle collisions (n=2), motorcycle collisions (n=2), falls (n=6), and assaults (n=2).

**Table 1:** Demographic characteristics.

		Healthy (n=132)	mTBI^a^ (n=12)
Age (years), mean (SD)		36 (10.2)	54.1 (22.3)
Sex, n (%)			
	Female	88 (67)	4 (33)
Race or ethnicity, n (%)			
	White	84 (64)	7 (58)
	Asian	24 (18)	1 (8)
	Black	12 (9)	2 (17)
	Hispanic	8 (6)	2 (17)
	Other	4 (3)	0 (0)
GCS^b^, median		15	15^c^

^a^mTBI: mild traumatic brain injury.

^b^GCS: Glasgow Coma Scale.

^c^One subject had a GCS of 14.

### Results of Statistical Analysis

Sample healthy and mTBI PLR curves produced by the smartphone app are shown in Multimedia Appendix 2. Significant differences were observed in PLR parameters of minimum diameter (*P*=.004), percent change, maximum diameter, and mean constriction velocity (*P*<.001; Table 2).

In the binary classification analysis, the SMOTE [17] produced a sample size of 132 mTBI PLR recordings and 132 healthy PLR recordings. The best-performing feature combinations based on AUC value across the 4 model types are listed in Table 3. The best-performing model overall was random forest, with the latency, percent change, minimum diameter, maximum diameter, mean constriction velocity, and maximum constriction velocity PLR parameters used as features. After stratified 10-fold cross-validation, this model produced an overall accuracy of 93.5%, sensitivity of 96.2%, specificity of 90.9%, AUC of 0.936, and *F*_1_-score of 93.7% for differentiating between PLR curves of mTBI and healthy cohorts.

**Table 2:** Smartphone pupillometry PLR^a^ parameters in healthy and participants with mTBI^b^.

PLR parameters	Healthy, mean (SD)	Acute mTBI, mean (SD)	*P* value
Latency (s)	0.21 (0.075)	0.19 (0.12)	.17
Percent change (%)	34 (8.3)	26 (7.9)	<.001
Minimum pupillary diameter (pixels)	34.8 (6.1)	29.7 (6.1)	.004
Maximum pupillary diameter (pixels)	53.6 (12.4)	40.9 (11.9)	<.001
Mean constriction velocity (pixels/s)	11.5 (5.0)	6.8 (3.0)	<.001
Max constriction velocity (pixels/s)	48.9 (20.5)	38.7 (28.8)	.06
Mean dilation velocity (pixels/s)	5.4 (2.3)	3.9 (2.1)	.02

^a^PLR: pupillary light reflex.

^b^mTBI: mild traumatic brain injury.

**Table 3:** Best performing binary classification models^a^.

Model	PLR^b^ parameter combination	Accuracy, %	Sensitivity, %	Specificity, %	AUC^c^	*F*_1_-score, %
RF^d^	Latency, percent change, maximum diameter, minimum diameter, mean constriction velocity, and maximum constriction velocity	93.5	96.2	90.9	0.936	93.7
KNN^e^	Percent change, maximum diameter, and minimum diameter	91.7	94.7	88.8	0.918	91.9
SVM^f^	Percent change, minimum diameter, mean constriction velocity, and mean dilation velocity	86	91	81	0.86	86.7
LR^g^	Maximum diameter, mean constriction velocity, and mean dilation velocity	86.3	95.5	77.4	0.864	87.7

^a^The absolute values are unable to be provided for the performance percentages reported here due to the mechanism of 10-fold cross-validation that was used to obtain them.

^b^PLR: pupillary light reflex.

^c^AUC: area under the curve.

^d^RF: random forest.

^e^KNN: k-nearest neighbors.

^f^SVM: support vector machine.

^g^LR: logistic regression.

## Discussion

### Principal Findings

We present data comparing PLR parameters (Textbox 1) in a cohort of patients with acute mTBI compared with healthy controls. Our results indicate that statistically significant differences can be detected between the mean PLR parameters of patients with acute mTBI and healthy controls using smartphone quantitative pupillometry. The percent change, minimum diameter, maximum diameter, and mean constriction velocity PLR parameters were significantly lower in the acute mTBI cohort (Table 2). This reflects the functional rather than structural abnormalities in neuronal homeostasis that are the basis of mTBI pathophysiology [19]. After using SMOTE [17] to resolve the class imbalance in our sample, we observed the performance of 4 binary classification models for differentiating between acute mTBI and healthy controls (Table 3), the best of which produced accuracy, sensitivity, specificity, AUC, and *F*_1_-score all above 90%, suggesting useful diagnostic discrimination.

### Comparison With Previous Work

There has been increased interest in PLR as a physiologic biomarker of mTBI and in automated pupillometry. One study of the NPi-200 commercial pupillometry device in patients with blast-induced mTBI 15-45 days post injury found that mean constriction velocity, latency, and mean dilation velocity were slower than controls [7]. A follow-up study of 100 soldiers with a concussion compared with 100 controls without a concussion <72 hours post injury had similar findings [20]. Pupillary changes have also been demonstrated in those with chronic mTBI compared with controls >45 days and >1 year post injury using automated quantitative pupillometry [8,9]. Most recently, changes in pupillary reactivity were demonstrated in 98 youths with a concussion compared with 134 controls at a median of 12 days post injury [10]. Smartphone apps have also been studied previously in the diagnosis and management of concussion and mTBI based on subjective clinical findings [21-23], although before this study, only 1 used pupillometry [24].

### Detailed Discussion of This Work

The smartphone pupillometer used in this study (PupilScreen) has several advantages over more traditional devices. It is more affordable and would be more accessible and practical in clinical care settings outside of the hospital. It also has demonstrated improved performance when compared with a proprietary pupillary reactivity index [25] in the setting of severe TBI [14], without effects from opioid medication use [15]. The smartphone pupillometer in this study has also shown potential use in the diagnosis of other neurological conditions such as in the detection of acute preintervention ischemic stroke while a proprietary pupil index [25] remained within the normal and reactive range for all participants who had stroke [13]. Other quantitative pupillometry technologies have been studied with varying hardware and software features and requirements [25-29], yet these technologies have not been studied as extensively, do not support simultaneous binocular recording of the PLR for dynamic assessment, and do not incorporate machine learning to uncover nuanced relationships between PLR parameters that may not be easily summarized in a proprietary reactivity index [25].

In this study, we observed alterations of the autonomic nervous system in mTBI compared with healthy controls (reduction in maximum and minimum pupil diameters) and direct effects of mTBI functional pathophysiology on cranial nerve III or its postganglionic short ciliary nerve derivatives [1] (difference in percent change and mean constriction velocity parameters). These results correlate with previous studies in acute mTBI [20] on the importance of the mean constriction velocity but not on that of the mean dilation velocity, which may be due to mechanical differences in the method of capture between other quantitative pupillometers and the smartphone quantitative pupillometer used in this study. A report of patients with chronic mTBI demonstrated findings similar to our study (despite evaluating chronic, rather than acute mTBI), finding significant differences seen in the maximum resting pupillary diameter, mean constriction velocity, maximum constriction velocity, mean dilation velocity, and percent change PLR parameters [8]. Our study is unique in that it includes only participants within 36 hours after injury, unlike others for which recruitment occurred up to several weeks after mTBI [7-10], and in that it uses smartphone pupillometry as an accessible and practical alternative to traditional quantitative pupillometry.

Using Multimedia Appendix 2 as an example, PLR curves between a healthy control and a patient with acute mTBI look subjectively similar to the naked eye. Despite this, a statistically significant difference was found in the structural curve morphology parameters listed above, indicating that using these quantitative PLR parameters in combination (rather than each one alone) may be necessary to detect subtle changes that may be present in acute mTBI. The results of our binary classification models support this, as when the PLR parameters are used in combination with one another as features in a machine learning binary classification model, we see a reasonable capability of the model to differentiate between healthy and participants with acute mTBI with more than 90% on all model performance metrics. In addition, the important PLR parameters mirror those from the literature and our individual parameter comparison results. While preliminary, our results show promise in the usage of a mobile smartphone pupillometer with advanced PLR analysis to detect mTBI, which could have major implications in fields such as athletics, prehospital care, the military, and digital health in general. Although we did not evaluate the diagnostic spectrum of mild, moderate, and severe TBI in this pilot study, such work is ongoing using the smartphone pupillometer studied here. In addition, we believe that there is value in studying an objective tool for acute mTBI differentiation from healthy controls as it has been demonstrated in the literature that cases of acute mTBI are missed in the acute care setting (such as the emergency department setting where this study was conducted) [30,31].

### Limitations

This study is limited by multiple factors, the first of which is the small sample size of 12 patients with acute mTBI. We have addressed this limitation through our use of SMOTE [17] to equalize the sample size of both cohorts to 132 recordings for binary classification machine learning analysis, nonetheless, larger studies are required for external validation and there is a risk of overfitting in the machine learning models when using this approach. Another limitation of this approach is the possibility that the sample of patients with acute mTBI is not representative of the broader acute mTBI population. Using the case descriptions in Multimedia Appendix 1, a heterogeneous distribution of case types is seen with a wide range in time after injury, a variety of mechanisms (falls, assaults, and motor vehicle collisions), and findings on examination that are qualifying for the ACRM definition of acute mTBI. Thus, we believe that despite the small sample size, we have captured a somewhat representative group of the broader emergency department population with acute mTBI using availability sampling. Another limitation is the mechanism of injury, which was entirely mechanically induced, which may limit the application of our findings to participants with blast-induced injury in military settings [7]. Finally, our healthy cohort was younger than the acute mTBI cohort, and thus known changes in the PLR along the spectrum of aging [32] may have affected our results.

### Conclusions

In this pilot study, mobile pupillometry using a smartphone app detected significant differences in PLR parameters and performed with greater than 90% accuracy, sensitivity, specificity, AUC, and *F*_1_-score on binary classification between acute mTBI and healthy cohort. The technology studied in this pilot study may have potential future use in hospital or nonhospital settings to detect acute mTBI and concussion after future validation to test the generalizability and stability of its predictions on prospectively collected external testing data sets.

## Appendix

Multimedia Appendix 1 Table – Injury Characteristics.

Multimedia Appendix 2 Acute mTBI (A) and healthy subject (B) pupillary light reflex (PLR) curves. Top panel: PLR curve of right (red) and left (blue) eyes. Bottom panel: Brightness of the recording as detected by the smartphone camera. Although some motion artifact is present in both curves, the mTBI and healthy subject curves appear qualitatively similar with pupillary constriction during increased brightness (due to the light stimulus from the smartphone camera flash) and pupillary re-dilation towards baseline diameter after cessation of light stimulus. Brightness is a unitless measurement of the ambient brightness detected by the built-in iPhone camera during the entire recording of the PLR. It is reported in APEX (Additive System of Photographic Exposure) which is an iPhone-specific measurement; more details can be found in iPhone software documentation.

## Abbreviations

ACRM: American College of Rehabilitation Medicine
AUC: area under the curve
FDA: United States Food and Drug Administration
mTBI: mild traumatic brain injury
PLR: pupillary light reflex
SMOTE: synthetic minority oversampling technique
TBI: traumatic brain injury
