# Supplementary material for: Smartphone Pupillometry and Machine Learning for Detection of Acute Mild Traumatic Brain Injury: Cohort Study
Source: JMIR Neurotechnol. 2024 Jun 13;3:e58398. doi: 10.2196/58398 (PMC12671303; doi:10.2196/58398)
Supplement: Multimedia Appendix 1 [file neuro_v3i1e58398_app1.docx]

| Case | Mechanism of injury | LOC (duration)? | LOC less than 30 minutes? | Loss of memory surrounding event? | Post-traumatic amnesia, less than 24 hours in duration? | Altered mental state immediately following injury? | Focal neurologic deficits? | GCS | Time from injury to PLR recording (hours) | Head CT findings without acute intracranial abnormality? |
| --- | --- | --- | --- | --- | --- | --- | --- | --- | --- | --- |
| 1 | MVC | Yes* | Yes | Yes | Yes | Yes | None | 15 | 29 | Yes |
| 2 | MVC | None | - | Yes | Yes | Yes | None | 15 | 4 | Yes |
| 3 | GLF | None | - | Yes | Yes | Yes | None | 15 | 0.5 | Yes |
| 4 | GLF | None | - | Yes | Yes | Yes | None | 15 | 17 | Yes |
| 5 | GLF | None | - | No | - | Yes | None | 15 | 12 | Yes |
| 6 | Fall (30 stairs) | None | - | Yes | Yes | Yes | None | 14 | 1 | Yes |
| 7 | Fall (10-foot ladder) | Yes* | Yes | Yes | Yes | Yes | None | 15 | 2 | Yes |
| 8 | MCC | Yes* | Yes | Yes | Yes | Yes | None | 15 | 1 | Yes |
| 9 | Assault | Yes* | Yes | Yes | Yes | Yes | None | 15 | 2 | Yes |
| 10 | Assault | Yes* | Yes | Yes | Yes | Yes | None | 15 | 10 | Yes |
| 11 | Fall (3 steps) | Yes (3 minutes) | Yes | Yes | Yes | Yes | None | 15 | 2 | Yes |
| 12 | MCC | Yes* | Yes | No | - | Yes | None | 15 | 1 | Yes |

LOC: loss of consciousness, MVC: motor vehicle collision, GLF: ground level fall, MCC: motorcycle collision, *Exact duration unknown
