# Supplementary figures and images for: Smartphone Pupillometry and Machine Learning for Detection of Acute Mild Traumatic Brain Injury: Cohort Study
Source: JMIR Neurotechnol. 2024 Jun 13;3:e58398. doi: 10.2196/58398 (PMC12671303; doi:10.2196/58398)

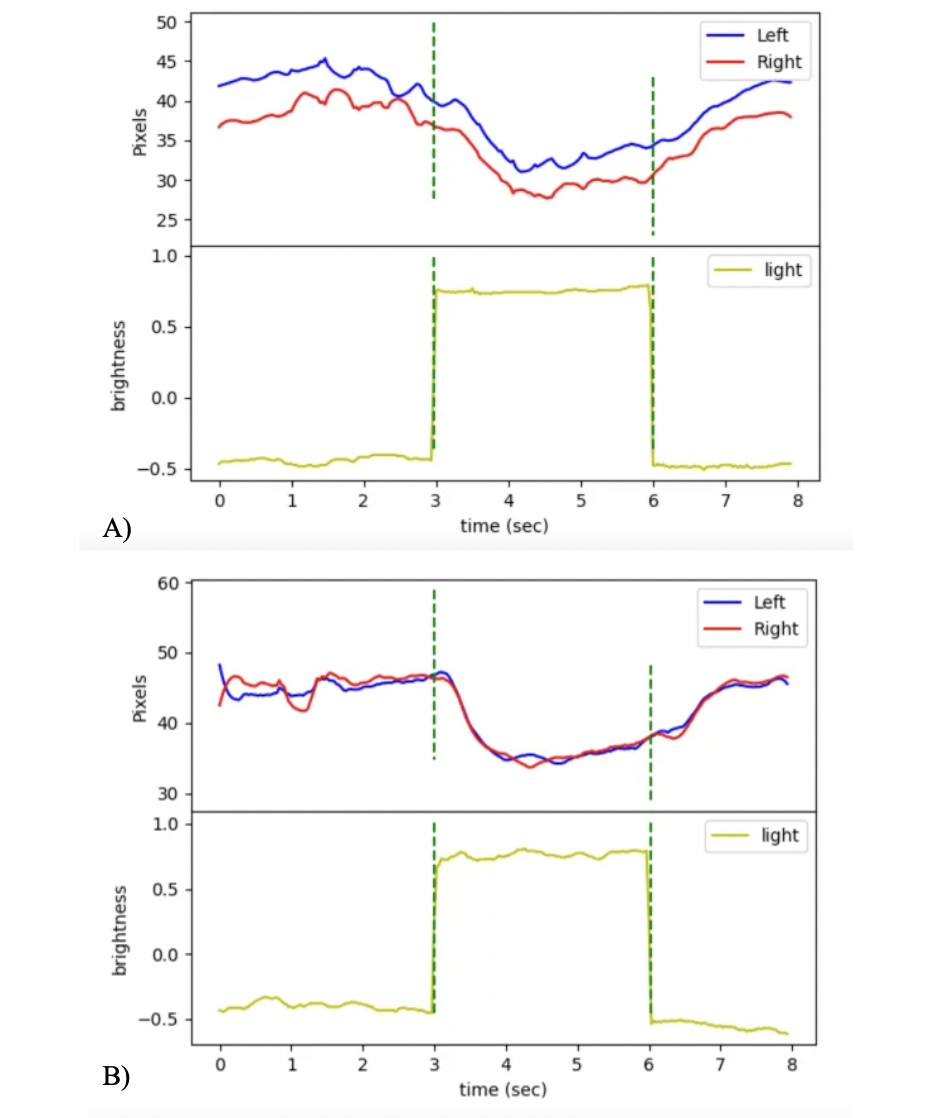

Supplement: Multimedia Appendix 2 [file neuro_v3i1e58398_app2.png]
